# Novel weight loss diet attenuates dietary-induced obesity in mice and might correlate with altered gut microbiota and metabolite profiles

**DOI:** 10.3389/fnut.2022.987955

**Published:** 2022-11-11

**Authors:** Xinli Yang, Li Bao, Ying Zhang, Jianglan Long, Yan Li, Huijun Wang, Yan Cui, Dan Yan

**Affiliations:** ^1^Department of Clinical Nutrition, Beijing Friendship Hospital, Capital Medical University, Beijing, China; ^2^Department of Pharmacy, Beijing Shijitan Hospital, Capital Medical University, Beijing, China; ^3^Beijing Key Laboratory of Bio-Characteristic Profiling for Evaluation of Rational Drug Use, Beijing, China; ^4^Department of Pharmacy, Beijing Friendship Hospital, Capital Medical University, Beijing, China; ^5^Department of Central Laboratory, National Institute for Nutrition and Health, Chinese Center for Disease Control and Prevention, Beijing, China; ^6^Department of Public Nutrition, National Institute for Nutrition and Health, Chinese Center for Disease Control and Prevention, Beijing, China; ^7^Institute for Infectious Disease and Endemic Disease Control, Tongzhou District Center for Disease Prevention and Control, Beijing, China; ^8^Department of Pharmacy, Beijing Institute of Clinical Pharmacy, Beijing, China; ^9^Beijing Friendship Hospital, Capital Medical University, Beijing, China

**Keywords:** obesity 肥胖症, dietary pattern 饮食习惯, body composition 身体成分, inflammatory factors 炎症因子, triglycerides, triglyceride 甘油三酯, gut microbiota 肠道菌群, fecal metabolites 粪便代谢物

## Abstract

Although many dietary patterns have been studied for weight loss, various limitations still exist. Therefore, we designed a novel weight loss diet (NWLD) with carbohydrate, protein, and fat (energy) contents of 45%, 20%, and 35%, respectively. The saturated fatty acids: monounsaturated fatty acids:polyunsaturated fatty acids ratio was 1:2:1, and the insoluble: soluble dietary fiber ratio was 2:1. We aimed to observe the effect of NWLD on weight loss and understand the underlying metabolic mechanisms. Twenty-nine male C57BL/6J mice were selected. Nine mice were fed ordinary feed in a blank control group, and the rest were fed a high-fat diet (HFD) to establish obese mouse models. Twelve weeks later, obesity models were established, and 10 obese mice were switched to NWLD feeding. Six weeks after switching the diet, the serum, intestinal feces, and kidneys of mice were collected. Obesity-related indicators, gut microbial composition, and fecal metabolite profiles of all the mice were determined, and the correlations among these indicators were analyzed. Kidney function indicators were also assessed. The results showed that the NWLD attenuated HFD-induced weight gain, serum triglycerides (TG), and inflammatory factors, optimized the body composition without kidney function impairment. Amino acid metabolism pathways and metabolites might play key roles in this process. The findings of this research imply that NWLD could be an effective nutritional remedy for managing dietary-induced obesity.

## Introduction

Undeniably, obesity poses a severe public health concern worldwide. Being overweight and obese are risk factors for several diseases, for example, hypertension, cancer, and cognitive impairment (1–3).

Dietary management is a critical component of obesity control strategies. The effects of multiple single foods or nutrients, such as dietary fiber (4) and symbiotic supplements (5), that can contribute to weight loss have been previously investigated. Distinct types of diets have also been developed based on the health effects of complex diets and dietary patterns (6); For instance, the low-carbohydrate diet, a diet with a carbohydrate (CHO) energy ratio of ≤ 45% of the total energy (7, 8), or even lower (9, 10). High-protein diets refer to diets with a percentage of energy-yielding protein >20% (11), >25% (12), or between 25% and 35% (13, 14). Other diets include vegetarian (15, 16) and vegan diets (17, 18), and the Mediterranean diet (19); some are high in protein but normal in CHO (20). However, these dietary patterns are not specifically defined and additionally, have limitations. For instance, various complications, such as heart arrhythmias, sudden death, and kidney damage, are linked to a long-term restriction of CHO in the diet (21). A high-protein diet may damage kidney function in obese patients (22), and adherence to vegetarian diets often lead to nutritional deficiencies (23, 24). The Mediterranean diet is also reportedly beneficial for weight loss (25); however, it is not specifically designed for weight loss. Chinese research showed that a low-fat and relatively high-CHO diet, similar in macronutrient composition to that traditionally consumed in China, is seemingly less likely to fuel excessive weight gain (26). However, according to the Report on **(author?)** (27), the percentage of energy-yielding dietary fat in Chinese diets has reached 36% in urban areas and 33.7% in rural areas. Modern lifestyles and dietary patterns substantially differ from those in the past. A sudden regress to the previous dietary patterns is unlikely. In addition, the application of advanced techniques, such as 16S ribosomal RNA (16S rRNA) sequencing, metagenomic analyses, and metabolomics revealed that the microbial composition not only could change rapidly in response to changes in the host diet or commonly used drugs (28, 29) but also have many significant associations with between long-term dietary information (30). Different types of weight-loss diets, such as high protein (30% calorie intake) (31), low-energy diets (800 and 1,200 kcal/day) (32), and the green-Mediterranean (33), have different effects on the gut flora. A caloric restriction intervention with fiber showed sex-specific effects on either adiposity and fasting insulin. These effects were thought to be linked to changes in specific gut microbiota species, functional genes, and bacterially produced metabolites (34). Higher-fat consumption by healthy young adults whose diet is in a state of nutrition transition appeared to be associated with unfavorable changes in gut microbiota, fecal metabolomic profiles, and plasma proinflammatory factors (35).

Considering the above-mentioned factors, we designed a novel weight loss diet (NWLD) that accommodates the current dietary habits in China. It is characterized by a macronutrient composition of 20% protein, 35% fat, and 45% CHO with a high dietary fiber content (insoluble to soluble, 2:1) and a ratio of saturated:monounsaturated:polyunsaturated fatty acids of 1:2:1. Our main objective was to test the functionality of the NWLD. The secondary objective was to preliminarily explore its underlying metabolic mechanisms, including the roles of the gastrointestinal flora and metabolism. We hypothesized that the NWLD affects metabolites via alterations in the gut microbiota, subsequently influencing obesity-related indicators. This hypothesis was evaluated in a murine model of obesity.

## Materials and methods

### Experimental design and sample collection

Twenty-nine normal, specific pathogen-free C57BL/6J male mice (6-week-old), purchased from Beijing Vital River Laboratory Animal Technology Co., Ltd. (Beijing, China; license number: SCXK (Beijing) 2012-0006), were housed in the animal facility of the Beijing Shijitan Hospital, Capital Medical University. They were kept at 24 ± 2°C, 45–60% relative humidity, 12 h-day/12 h-night cycles (light phase 06:30–18:30), with *ad libitum* access to standard food and water for 1 week before the experiments. Then, mice were randomly divided into two groups using simple randomization, with random numbers generated with the standard = RAND() function in Microsoft Excel (36). The control group comprised normal mice (NC group, *n* = 9) fed standard fodder, while model mice (*n* = 20) were fed an high-fat diet (HFD) (feed formula in Table 1) for some weeks to induce obesity. Then, model mice were divided into HFD and NWLD groups (*n* = 10 per group) using simple randomization. Mice in the HFD group were fed an HFD, while those in the NWLD group were fed the NWLD (Table 1).

**Table 1:** Three mouse feed formulations.

	**NWLD**		**NC (Standard Fodder)**		**HFD**	
	**Mass percentage**	**Energy percentage**	**Mass percentage**	**Energy percentage**	**Mass percentage**	**Energy percentage**
**Protein**
Casein	22.40%	20.00%	20.00%	20.00%	25.80%	20.00%
L-cystine	0.30%		0.30%		0.40%	
**CHO**
Corn starch	27.10%	45.00%	39.70%	64.00%	0.00%	20.00%
Maltodextrin	11.70%		13.20%		16.20%	
Sucrose	8.00%		10.00%		8.90%	
Cellulose	5.50%		5.00%		6.50%	
Inulin	2.80%		–		–	
**Fat**
Olive oil	7.10%	35.00%	–	16.00%	–	60.00%
Linseed oil	3.60%		–		–	
Peanut oil	2.90%		–		–	
Beef tallow	4.00%		–		–	
Soybean oil	–		7.00%		3.20%	
Lard	–		–		31.70%	

NC, normal control group; HFD, high-fat diet group (model); NWLD, novel weight loss diet group; CHO, carbohydrate; –, this substance was not added in the diet.

During the experiments, food and water were provided *ad libitum*, and conditions in the animal facility were kept constant. All mice survived the whole experimental phase. The serum and intestinal contents, and kidneys were collected from each animal. The body weights of all 29 mice were measured every 2 weeks. At the end of the treatment period, five mice in each group were randomly selected for body composition analyses, after which all mice were fasted for 12 h, anesthetized, and sacrificed by cervical dislocation after weighing. The animal care procedures and methods adopted were approved by the Animal Care and Use Committee of the Scientific Research Ethics Committee, Beijing Shijitan Hospital, Capital Medical University (permission number: 2017-035). All possible efforts were made to minimize the number of animals used and their suffering.

Six weeks after the diet change, mice were sacrificed (with CO_2_ inhalation) to collect the full blood, kidneys, jejunum, and ileum. Whole blood samples were collected through eyeball extirpation. The serum was prepared as follows: whole blood was left at room temperature for 60 min and then centrifuged (3,500 rpm for 15 min) to remove remaining insoluble residuals. The serum was then stored at −80°C. Next, jejunum and ileum were collected, divided into 250 mg per tube, and immediately treated with liquid nitrogen for 15 min. After labeling, these samples were stored at −80°C and then thawed in a refrigerator at 4°C for measurement. Kidneys were collected and weighted. The serum, feces, and kidneys were the final samples we tested.

### Laboratory and statistical analysis

The standard fodder was purchased from Beijing HFK Bioscience Co., Ltd. (Beijing, China), and the high-fat feed from Research Diets, Inc. (New Brunswick, NJ, USA). To analyze blood samples, we used the Blood Lipid Series kit (Nanjing Jiancheng Biotechnology Co., Ltd., Nanjing, China). Inflammatory factors were detected using an ELISA kit (Beijing Solarbio Science & Technology Co., Ltd., Beijing, China). A microplate reader was provided by the Beijing Institute of Clinical Medicine (Beijing, China). Serum creatinine and urea were tested by fully automatic biochemical instrument BS-420 produced by MINDRAY Bio Medical Electronic Limited by Share Ltd. Body composition was analyzed using a 7 Tesla animal Magnetic resonance imaging scanner (7.0/16, Agilent, US) at the Imaging Center of the Institute of Medical Laboratory Animals, Chinese Academy of Medical Sciences. Sequencing was outsourced to the Wekemo Tech Group Co., Ltd. (Shenzhen, China). Data were analyzed using the free online platform Wekemo Bioincloud.

SAS software v.9.2 (SAS Institute, Cary, NC, USA) and GraphPad Prism 6.2 (GraphPad Software, San Diego, CA, USA) were used to analyze phenotypic characteristics and generate plots. R Programming Language and Software and GraphPad Prism 6.2 were used to analyze the microflora and metabolomics data and generate plots. Student's *t*-test for independent samples was used for comparisons between two groups, and one-way analysis of variance (ANOVA) and Bonferroni correction were used to compare multiple groups. The results were considered significant at a level of *p* < 0.05.

### Phenotypic characteristics

Phenotypic characteristics such as body weight, blood lipid levels, inflammatory factors, and body composition were measured. Serum triglyceride (TG), total cholesterol (TC), HDL cholesterol, LDL cholesterol, high-sensitivity C-reactive protein (hs-CRP), interleukin-6, interleukin-1β (IL-1β), and tumor necrosis factor-α (TNF-α) levels were assessed with a microplate reader (Molecular Device, SpectraMax M3) according to the manufacturer's instructions.

During *in vivo* imaging, mice were anesthetized with 2% isoflurane in pure O_2_, and the isoflurane was reduced to 1% for the maintenance of anesthesia. MRI studies of this line of mice were conducted on the 7 Tesla MRI system. The SE-T1WI imaging parameters were as follows: TR = 665 ms, TE = 13 ms, FOV = 96 × 58 mm, Thk = 0.6, matrix = 256 × 256, and NEX = 8. We calculated the volume of total fat (T-fat), total muscle, subcutaneous fat (SCF), visceral fat (VF), and brown fat (BF), and the percentage of different types of fat in T-fat volume, including SCF percentage (SCF%), VF percentage (VF%), and BF percentage (BF%).

To assess kidney function in mice, we also calculated their renal index (kidney weight as a percentage of body weight) except for serum creatinine and urea.

### Fecal analysis

#### 16S rRNA gene sequencing analysis

Microbial DNA was extracted from cecal fecal samples using the E.Z.N.A.^®^ soil DNA Kit (Omega Bio-Tek, Norcross, GA, U.S.) according to the manufacturer's protocols. The V3–V4 hypervariable regions of the bacterial 16S rRNA gene were amplified with primers 341F (5′-CCTAYGGGRBGCASCAG-3′) and 806R (5′-GGACTACNNGGGTATCTAAT-3′) by thermocycler PCR system (GeneAmp 9700, ABI, USA). The detailed procedure is described in the Supplementary material. The analysis was conducted following the “Atacama soil microbiome tutorial” of Qiime2docs, along with customized program scripts (https://docs.qiime2.org/2019.1/). The QIIME2 dada2 plugin was used to obtain the feature table of the amplicon sequence variant (37). The QIIME2 feature-classifier plugin was then used to align amplicon sequence variant sequences to a pre-trained GREENGENES 13_8 99% database (trimmed to the V3–V4 region bound by the 338F/806R primer pair) to generate the taxonomy table (38). Any contaminating mitochondrial, chloroplast, chimera, and low-quality sequences were filtered using the QIIME2 feature-table plugin. Appropriate methods, including ANOVA, Kruskal–Wallis, linear discriminant analysis (LDA) effect size (LEfSe), and DEseq2, were employed to identify differentially abundant bacteria between samples and groups (39–41). Diversity metrics were calculated using the core-diversity plugin within QIIME2. Feature level alpha diversity indices, such as observed operational taxonomic units (OTUs), Chao1 richness estimator, Shannon diversity index, and Simpson index, were calculated to estimate the microbial diversity within an individual sample. Weighted UniFrac, a beta diversity distance measurement, was performed to investigate the structural variation of microbial communities across samples and then visualized via principal component analysis (42). Co-occurrence analysis was performed by calculating Spearman's rank correlations between predominant taxa, and the network plot was used to display the associations among taxa. The correlation network was drawn by the R language igraph package. In addition, the potential Kyoto Encyclopedia of Genes and Genomes (KEGG) Ortholog functional profiles of microbial communities were predicted using Phylogenetic Investigation of Communities by Reconstruction of Unobserved States (43). Unless specified above, parameters used in the analysis were set as default.

#### Fecal metabolic analysis

For metabolite extraction, 50 mg mouse feces samples were accurately weighed, and the metabolites were extracted using a 400 μl methanol:water (4:1, v/v) solution. The mixture was allowed to settle at −20°C and treated with a high-throughput tissue crusher Wonbio-96c (Shanghai Wanbo Biotechnology Co., Ltd.) at 50 Hz for 6 min, followed by vortexing for 30 s and ultrasound treatment at 40 kHz for 30 min at 5°C. Samples were placed at −20°C for 30 min to precipitate proteins. After centrifugation at 13,000 rpm at 4°C for 15 min, the supernatants were carefully transferred to sample vials for liquid chromatography-mass spectrometry/mass spectrometry analysis. The detailed procedure is described in the Supplementary material. A multivariate statistical analysis was performed using ropls (V. 1.6.2) R package from Bioconductor on Majorbio Cloud Platform (https://cloud.majorbio.com). The machine learning algorithm support vector machine (SVM) was used for discriminant analysis to calculate the average importance of metabolites with significant differences between groups. To render the data close to a normal distribution, the normalization function (with the arguments MedianNorm, LogNorm, and AutoNorm) was adopted. Significant metabolites were evaluated by a KEGG pathway enrichment analysis to identify related metabolic pathways. Fold change values were calculated to measure changes in metabolites. A volcano plot was used to filter metabolites of interest based on log_2_ (fold change) and –log_10_ (*p*-value). The metabolites with *p* < 0.05 (*t*-test) were used to conduct an over-representation analysis (ORA), and the resulting KEGG pathways with *p* < 0.05 (ORA) were considered statistically significantly enriched. Topology analysis was performed to determine which pathways had a greater impact; a higher impact value indicated that the metabolites were more closely connected to upstream factors, playing a greater role in observed differences between groups. Support vector machine was implemented in the R package the MetaboAnalystR (44). The normalization function is included in the MetaboAnalystR package. Volcano plots were visualized with the ggplot2 package in R language.

## Results

### General condition

After 12 consecutive weeks, the average weight of model mice fed an HFD was 20.8% higher than that of NC animals. There was a significant difference (*p* < 0.0001) in body weight between the two groups, indicating that the obesity model was successfully established. The daily feed intake for each group was stable throughout the experimental period. During the experiment, mice in the NC group exhibited good activity and normal feeding, whereas those in the HFD group were lethargic. After 10 weeks, mice in the HFD group showed different degrees of activity reduction. Furthermore, the body weight of mice in the HFD group increased during the first 4 weeks and decreased slightly over the following 2 weeks. By contrast, the body weight of mice in the NWLD group decreased steadily and was similar to that of mice in the NC group by week 6 (Table 2). The mean body weight of mice in the model group was 20.8% (5.42 g) higher than that of mice in the NC group (week 0). There was a statistically significant difference in body weight between mice in the NWLD and HFD groups after 4 weeks of intervention. Furthermore, the weight loss in the NWLD group was significant within 6 weeks of intervention (from 30.86 to 27.30 g, an 11.5% decrease).

**Table 2:** Body weight (g) changes of mice fed with different diets (mean ± SD).

**Groups**	**NC**	**HFD**	**NWLD**
Week 0	26.00 ± 1.30	31.48 ± 3.10	30.86 ± 1.42
Week 2	26.21 ± 1.17	33.14^*^± 3.83	27.78 ± 1.36
Week 4	27.52 ± 1.24	35.22^*^± 4.80	28.09^#^ ± 1.36
Week 6	27.34 ± 1.58	33.33^*^± 3.15	27.30^#^ ± 0.99

NWLD, novel weight loss diet group.

* There were significant differences with group NC, normal control group.

# There were significant differences with group HFD, high-fat diet group (model).

### Phenotypic characteristics

**Figure 1:**
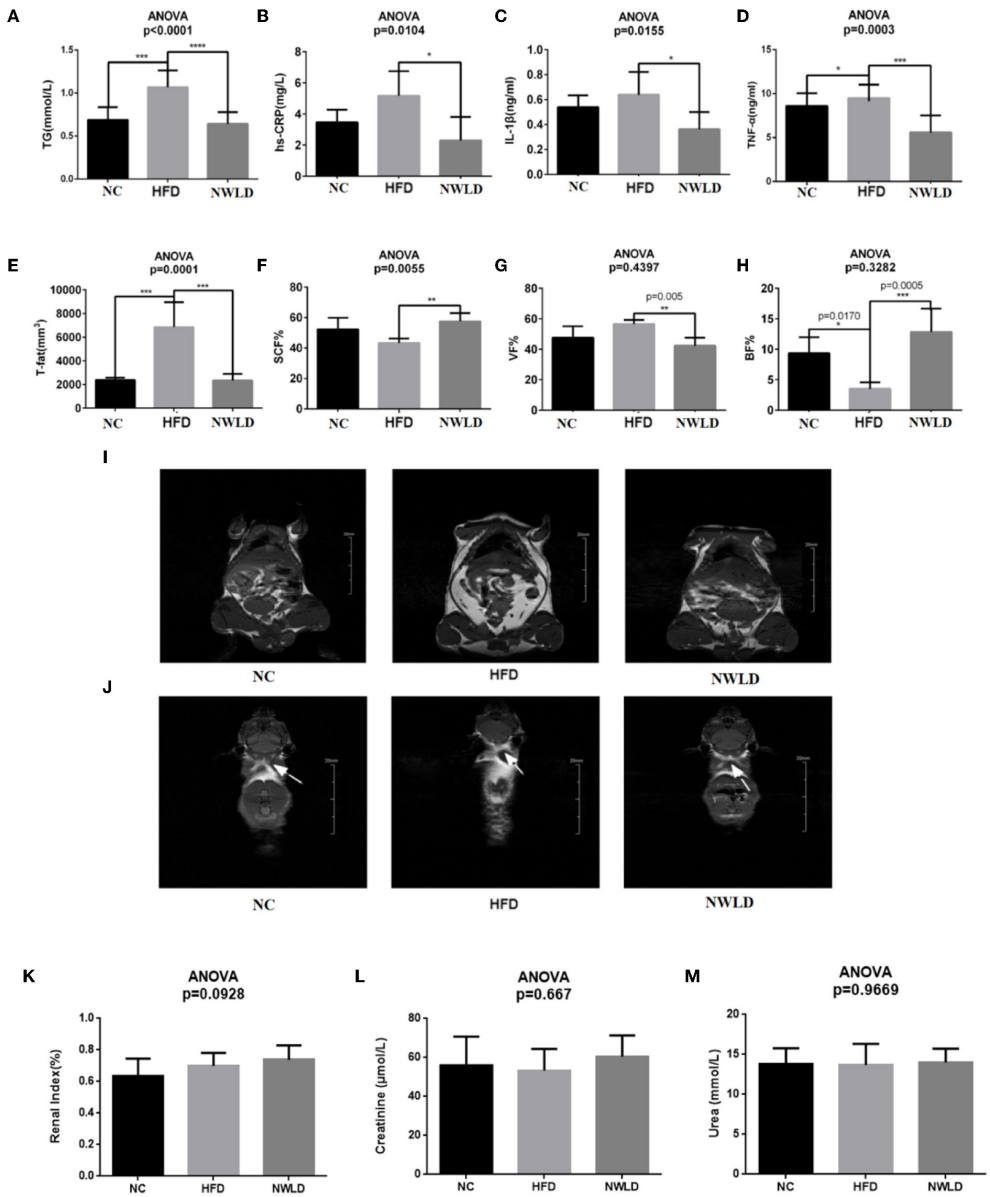
Comparison of phenotypic factors among groups. Bar charts of **(A)** serum triglycerides (TG), **(B)** hs-CRP, **(C)** IL-1β, **(D)** TNF-α, **(E)** total fat, **(F)** subcutaneous fat percentages, **(G)** visceral fat percentages, **(H)** brown fat percentages, and **(I)** white fat (the highlighted areas in the abdomen and subcutaneous tissue) in each group (coronal plane). Images were taken from NC4 section 7, HFD3 section 9, and NWLD3 section 6. **(J)** Brown fat of mice in different groups (butterfly areas are indicated by arrows). Images were taken from NC2 section 8, HFD5 section 29, and NWLD4 section 29. *p < 0.05; **p < 0.01; ***p < 0.001; ****p < 0.0001. Bar charts of **(K)** renal index, **(L)** serum creatinine, **(M)** serum urea.

As shown in Figures 1A–M, in terms of plasma lipids, TG levels were the lowest in the NWLD group (0.64 ± 0.13 mmol/L) and highest in the HFD group (1.07 ± 0.2 mmol/L), differing significantly from levels in the other two groups. Regarding inflammatory indicators, the hs-CRP level in the HFD group (5.17 ± 1.59 mmol/L) was significantly higher than in the other two groups. IL-1β (0.36 ± 0.14 ng/ml) and TNF-α (2.3 ± 1.51 ng/ml) levels were significantly lower in the NWLD group than in the other two groups. Concerning body composition, T-fat levels in the HFD group were significantly higher than those in groups NC and NWLD, while VF% and SCF% were significantly lower in the NWLD than in the HFD group. By contrast, the SCF% was significantly higher in the NWLD group. The accumulation of SCF and VF in the HFD group was apparent (Figures 1I,J). There were no significant differences in kidney function indicators among the three groups (Figures 1K–M).

The improvements in obesity-related phenotypes in NWLD-fed mice were in line with our expectations without kidney function impairment. Therefore, we conducted further studies on gut microbiota and metabolomics of fecal bacteria to explore the potential underlying mechanisms.

### Fecal 16S rRNA gene sequencing analysis

#### Alternative gut microbiota composition in different groups

**Figure 2:**
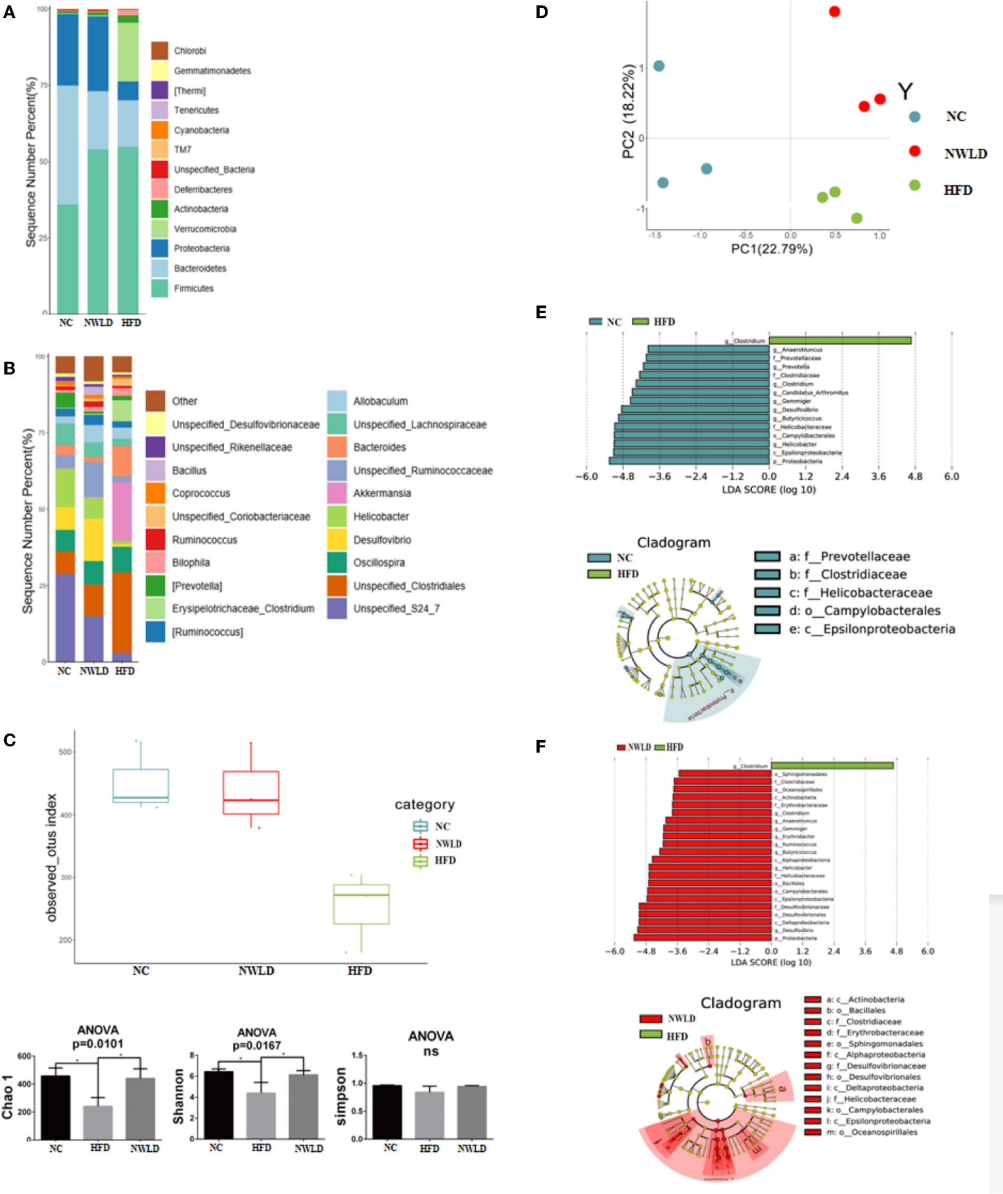
Characteristics of the gut microbiota in different groups. **(A)** Histograms of the relative distribution of groups at the phylum level (top 20 species in relative abundance); **(B)** histograms of the relative distribution of groups at the genus level (top 20 species in relative abundance); **(C)** alpha diversity. Box plots of observed OTUs, histogram of Chao1, Shannon, and Simpson indices. ANOVA, *p < 0.05; ns, no significance. **(D)** Beta diversity; **(E)** LEfSe analysis LDA histograms (LDA score >2.0) and cladogram of characteristic microorganisms (the NC group compared to group HFD). Kruskal–Wallis test, *p < 0.05. LDA, each transverse column represents a species; the length of the column represents the LDA score, where a higher LDA score indicates a greater difference. The color of the bar indicates the species group. Layers from the inside to outside of the cladogram correspond to different classification levels, i.e., kingdom, phylum, class, order, family, and genus, and the lines between levels represent relationships. Each circular node represents a species. Yellow nodes indicate no significant difference between groups; non-yellow nodes indicate that the species is a characteristic microorganism of the corresponding group (with significantly higher abundance in this group). The shaded fans mark the subclassification interval of the characteristic microorganism. **(F)** LEfSe analysis LDA histograms (LDA score >2.0) and cladogram of characteristic microorganisms (group NWLD compared to group HFD). Kruskal–Wallis test, *p < 0.05.

A total of 1,537 OTUs were detected in the public sequence of the GreenGenes repository. The gut microbiota characteristics of mice in each group are presented in Figure 2.

At the phylum level, the abundance of the top three species in the NC group was 39% (Bacteroidetes), 35% (Firmicutes), and 23% (Proteobacteria). Firmicutes/Bacteroidetes (F/B) ratio = 0.90. The abundance of Firmicutes, Verrucomicrobia, Bacteroidetes, and Proteobacteria in the HFD group was 55%, 19%, 15%, and 6%, respectively, F/B ratio = 3.67 (significantly different compared to the NC group, *p* < 0.05). The abundance of Firmicutes, Proteobacteria, and Bacteroidetes in the NWLD group was 54%, 24%, and 19%, respectively, F/B ratio = 2.84 (no significant difference compared to other groups).

At the generic level, the dominant genera in the NC group were *Unspecified_S24_7* (29%), *Helicobacter* (13%), *Oscillospira, Unspecified_Clostridiales, Desulfovibrio, Unspecified_Lachnospiraceae* (7%), and *Prevotella* (5%). The dominant genera in the NWLD group were *Desulfovibrio* (14%), *Unspecified_S24_7* (15%), *Unspecified_Ruminococcaceae* (11%), *Unspecified_Clostridiales* (10%), *Oscillospira* (8%), *Helicobacter* (7%). The dominant genera in the HFD group were *Unspecified_Clostridiales* (26%), *Akkermansia* (19%), *Bacteroides* (10%), *Oscillospira* (8%), and Clostridium (7%).

#### Alpha and beta diversity analysis

As determined by observed OTUs and Chao1 and Shannon indices (Figure 2C), the alpha diversity was significantly lower in the HFD group than in the NC group (*p* = 0.0495 for all comparisons) and was significantly higher in the NWLD than in the HFD group (*p* = 0.0495). The composition of the microbial communities among different samples was compared by a beta diversity analysis, and the results are summarized in Figure 2D.

#### Different abundance between groups of bacteria at the genus level

Compared to the NC and NWLD groups, the characteristic genus of the HFD group was *Clostridium*, which was from the Firmicutes phylum, *Erysipelotrichaceae family* (Figure 2D).

Compared to the HFD group, the characteristic bacteria of the NC group were *Gemmiger, Candidatus_Arthromitus, Butyricicoccus*, and *Anaerotruncus* (Firmicutes phylum); *Clostridium* (Firmicutes phylum, *Clostridiaceae family*); *Desulfovibrio* and *Helicobacter* (Proteobacteria phylum); *Prevotella* (Bacteroidetes phylum).

Compared with the HFD group, the characteristic bacteria of the NWLD group were *Gemmiger, Butyricicoccus, Anaerotruncus, Ruminococcus* (Firmicutes phylum); *Clostridium1* (Firmicutes phylum, *Clostridiaceae family*); *Erythrobacter, Helicobacter, Desulfovibrio* (Proteobacteria phylum).

### Results of fecal metabolic analysis

#### Identification of characteristic metabolites and key metabolites

**Figure 3:**
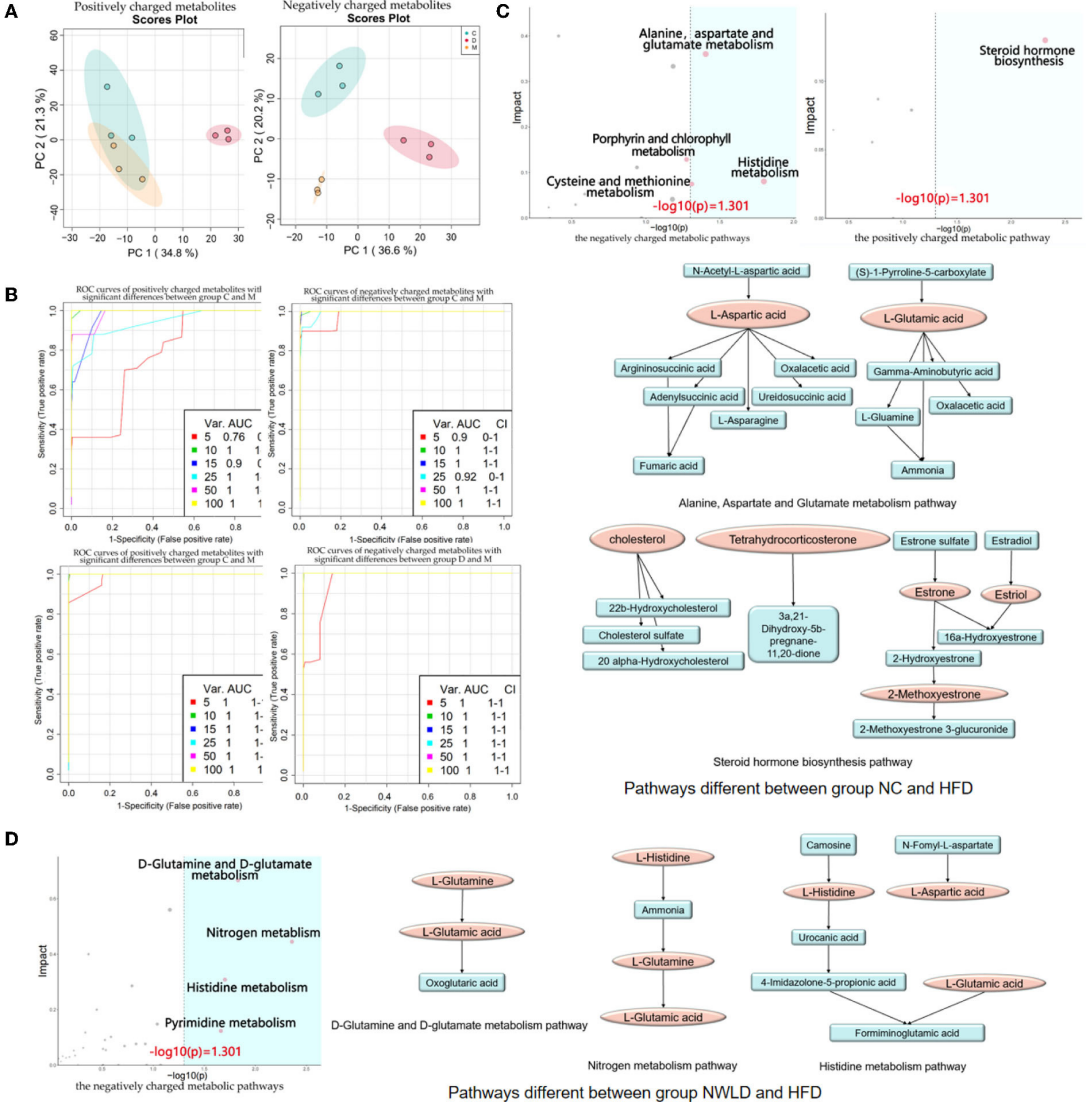
Fecal untargeted metabolomics results. **(A)** Principal component analysis score plots for discriminating the fecal metabolome (positive and negative charge metabolites) from the NC, HFD, and NWLD groups; **(B)** SVM ROC curves. The abscissa indicates accuracy, and the ordinate indicates sensitivity. AUC values closer to 1 indicate a better predictive accuracy and a greater difference in metabolites between groups; **(C)** ORA and topology analyses of metabolic pathways with significant differences between groups NC and HFD. The horizontal coordinate represents the ORA *p*-value, and the blue area is significant (*p* < 0.05). The vertical axis shows the impact value in the topology analysis. Maps of the main metabolic pathways are shown, including the alanine, aspartate, and glutamate metabolism pathways and steroid hormone biosynthesis; **(D)** ORA and topological analyses of the metabolic pathways with significant differences between groups NWLD and HFD and maps of the main metabolic pathways, including D-glutamine, D-glutamate, nitrogen, and histidine metabolism pathways.

We identified 707 differential metabolites between groups (*p* < 0.05), with 396 and 311 being positively and negatively charged, respectively. The most influential pathway was the amino acid metabolism pathway. Metabolite differences were concentrated in the comparisons between NC and HFD groups as well as between NWLD and HFD groups (but not between NC and NWLD groups). Differences in metabolite composition between groups, ORA, and topology analysis are summarized in Figure 3 and Table 3. In Figure 3, we showed the most important parts of the metabolic pathways, and the full contents of metabolic pathways are listed in Supplementary Figure 1.

**Table 3:** The metabolic pathways with significant enrichment of differential metabolites between groups and their ORA enrichment analysis and topo analysis.

**Metabolic pathway**	**KEGG pathway enrichment analysis**		**Topo analysis**
	**Fold enrichment**	**ORA *P*-value**	**Impact value**
**NC-HFD**
Alanine, aspartate, and glutamate metabolism	6.2149	0.0394	0.36
Steroid hormone biosynthesis	4.2784	0.0048	0.13
**NWLD-HFD**
D-glutamine and D-glutamate metabolism	9.9439	0.0147	0.67
Nitrogen metabolism	8.2865	0.0043	0.44
Histidine metabolism	4.9719	0.0199	0.31

The ORA (*p* < 0.05) indicated that the metabolites of interest were significantly enriched in these metabolic pathways. The degree of enrichment is expressed as the “fold enrichment.” A topology analysis illustrated the magnitude of the impact of significant pathways in the ORA. The greater the impact value, the greater the impact of the pathway; NC, normal control group; HFD, high-fat diet group (model); NWLD, novel weight loss diet group.

Differences in metabolites in the above pathways were analyzed using Student's *t*-tests. The most important differential metabolic pathways between the NC and HFD groups included the alanine, aspartate, and glutamate metabolism pathways and the steroid hormone biosynthesis pathway. The NWLD and HFD groups showed differences in the D-glutamine and D-glutamate metabolism, nitrogen metabolism, and histidine metabolism pathways. The key metabolites are shown in Figure 3C (between NC and HFD groups) and Figure 3D (between NWLD and HFD groups; marked in red).

As shown in Table 4, the mean peak areas for L-aspartic acid and L-glutamic acid were increased in the HFD group and decreased in the NWLD group compared to those in the NC group. L-aspartic acid levels, which were enriched in different pathways, negatively correlated with most of the aforementioned beneficial taxa. In addition, the fecal glutamate/glutamine ratio was higher in the NWLD (30.78) than in the HFD group (22.31). The model predictive performance was assessed by a receiver operating characteristic (ROC) curve. The areas under the curve (AUC) were all >0.72 (Figure 3B).

**Table 4:** The peak area of differential key metabolites compared between groups.

**Differential key metabolites**	**Group and group mean of peak area**	**Which-max**
**NC-HFD**
L-aspartic acid	NC 49.523, HFD 76.264	HFD
L-glutamic acid	NC 102.180, HFD 172.009	HFD
Cholesterol	NC 0.377, HFD 0.618	HFD
Tetrahydrocorticosterone	NC 0.492, HFD 1.732	HFD
Estriol	NC 0.345, HFD 0.782	HFD
Estrone	NC 6.622, HFD 0.286	NC
2-Methoxyesteone	NC 1.301, HFD 0.393	NC
**HFD-NWLD**
L-glutamine	HFD 7.711, NWDL 3.466	HFD
L-glutamic acid	HFD 172.009, NWDL 106.681	HFD
L-histidine	HFD 29.568, NWDL 8.474	HFD
L-aspartic acid	HFD 76.264, NWDL 38.785	HFD

NC, normal control group; HFD, high-fat diet group (model); NWLD, novel weight loss diet group.

#### Correlation network analysis

We selected gut microbial taxa and fecal metabolites with significant differences among dietary feeding groups to construct an interaction network. The results are presented in Table 5; the interaction network diagram is shown in Supplementary Figure 2.

**Table 5:** Result of Correlation Network Analysis among relative frequency of gut microbes, phenotype factors, and characteristic metabolites.

**Gut microbes**	**Phenotype factors**	**Correlation coefficient and *P-*value**	**Characteristic metabolites**	**Correlation coefficient and *P-*value**
**HFD**
*Clostridium*	Body weight	*r* = 0.94, *P* = 0.005	Tetrahydrocorticosterone	*r* = 0.89, *P* = 0.019
**NWLD**
*Butyricicoccus*
	TG	*r* = −0.94, *P* = 0.0048	L-aspartic acid	*r* = −0.94, *P* = 0.0048
	VF%	*r* = −0.83, *P* = 0.0416	L-glutamate	*r* = −0.83, *P* = 0.0416
	hs-CRP	*r* = −0.89, *P* = 0.0188		
*Desulfovibrio*
	TG	*r* = −0.94, *P* = 0.048	L-asparaguses	*r* = −0.94, *P* = 0.0048
	VF%	*r* = −0.83, *P* = 0.0416		
	BF%	*r* = 0.81, *P* = 0.0499		
*Erythrobacter*
	hs-CRP	*r* = −0.88, *P* = 0.0206		
	BF%	*r* = 0.95, *P* =0.003		
*Ruminococcus*
	TG	*r* = −0.83, *P* = 0.0416		
	VF%	*r* = −0.94, *P* = 0.0048		
*Gemmiger*
	hs-CRP	*r* = −0.81, *P* = 0.0499		
*Anaerotruncus*
	BF%	*r* = 0.84, *P* = 0.0361		

*P-*value < 0.05 were listed. NC, normal control group; HFD, high-fat diet group (model); NWLD, novel weight loss diet group; TG, total cholesterol; hs-CRP, high-sensitivity C-reactive protein; VF%, visceral fat percentage; BF%, brown fat percentage.

Regarding metabolites, L-glutamate was positively linked to hs-CRP (*r* = 0.89, *p* = 0.018) and IL-1β (*r* = 0.94, *p* = 0.0048). As a differential metabolite, L-histidine was enriched in histidine and nitrogen metabolic pathways. However, no correlation was found between L-histidine, gut microbiota, and obesity-related factors.

## Discussion

To avoid the disadvantages of previous weight loss diets, we designed the NWLD specifically for obese mice, taking into account weight loss, fat distribution, protection of kidney function, satiety stimulation, and compliance. We also combined the dietary habits prevalent in China. In this study, we established a mouse model of HFD-induced obesity, as evidenced by a decrease in BF% and significant increases in body weight, T-fat, VF%, TG levels, inflammatory factors, and a decrease in BF%. 16S rRNA gene sequencing and metabolomics analysis revealed a lower overall diversity in the gut microbiota of obese mice, with disrupted amino acid and lipid metabolism. In addition, interaction network analysis revealed that these microbial and metabolic parameters correlated with body weight, TG levels, inflammatory factors, and body fat distribution. The NWLD attenuated the HFD-induced changes in the microbiota and phenotype. Novel weight loss diet-fed mice had lower T-fat and VF% levels and higher SCF% and BF% than mice in other groups. These body composition and fat distribution patterns are associated with lower occurrence and progression rates of chronic diseases. An interaction network analysis was constructed to further understand the occurrence of HFD-induced obesity and elucidate the mechanisms underlying the beneficial effects of NWLD. Results showed that the obesity-related phenotypic factors correlated with specific changes in the flora and fecal amino acid metabolites, providing insight into the mechanism underlying the effects of the NWLD.

The composition of the intestinal microbiota is also closely related to host health. At the phylum level, our results have shown that HFD reduces gut microbial richness and increases the F/B ratio, which is supported by a previous study (45). We also found that the abundance and diversity of gut microbiota increased, and the F/B ratio decreased in the NWLD group. Previous studies have confirmed that *Bacteroidetes* primarily reside in the distal intestinal tract and are the main carbohydrate-degrading bacteria. They contain a variety of polysaccharides and glycosidases (46, 47) and participate in the fermentation of indigestible polysaccharides, such as dietary fibers like cellulose, hemicellulose, and β-glucan, to produce short-chain fatty acids (SCFAs; mainly acetate, butyrate, and propionate) (48). Short-chain fatty acids influence host metabolism in multiple ways by acting on G protein-coupled receptor 41 and G protein-coupled receptor 43. Acetate and butyrate subsequently induce the secretion of glucagon-like peptide 1 and peptide tyrosine-tyrosine, contributing to increased energy expenditure (49), reduced food intake (50), and improved glucose metabolism and insulin secretion (51). They can regulate the dynamic balance of adipose tissue by regulating the balance between anabolism and oxidation, which plays a key role in regulating obesity (52). In addition, at the genus level, we found that *Butyricicoccus, Ruminococcus, Desulfovibrio, Erythrobacter, Gemmiger*, and *Anaerotruncus* were negatively correlated with obesity-related risk factors and acted as beneficial bacteria. Among them, *Butyricicoccus* and *Ruminococcus* were relatively common butyric-producing bacteria (46). Short-chain fatty acids were not detected by untargeted metabolomics methods in our study. However, the correction of gut microbiota structure and the increased abundance of SCFAs-producing flora described above may explain the beneficial effects of NWLD.

It is known that intestinal metabolites are directly influenced by gut microbial composition and affect plasma metabolomics via absorption into the blood (53). One study showed that plasma aspartic acid was present at a significantly lower concentration in adult patients with obesity enrolled in a 3-week controlled body mass reduction program (54). Plasma metabolomics was not examined in our study. However, our results showed that in metabolites of fecal flora, L-aspartic acid increased in the HFD group and decreased in the NWLD intervention group, consistent with changes in plasma aspartic acid concentrations reported in a previous study. In addition, L-aspartic acid was inversely correlated with the aforementioned beneficial flora in our research. These results suggest that fecal L-aspartic acid may cause adverse effects. Experimental evidence shows that the circulating glutamate levels positively correlate with VF, abdominal obesity, and insulin resistance (55–57). The marked increase in plasma glutamate concentrations can cause a modest increase in plasma insulin concentration and induce fatty acid synthesis (58). Our results showed no direct correlation between fecal L-glutamate levels and VF%. However, the concentration of fecal glutamate increased in the HFD group, and the concentrations of fecal glutamate and glutamine were lower in the NWLD group, partially explaining the decrease in weight and T-fat levels in the NWLD-fed group. Histidine, mainly obtained from dietary sources, accounts for up to 15% of the metabolizable energy (59) and glucose production by gluconeogenesis (60). Thus, changes in histidine metabolism may lead to an imbalance in energy and glucose homeostasis. Recent metabolomics analyses of fasting plasma (61) and feces (62) have revealed that obesity is associated with disrupted histidine metabolism, consistent with the results of our research.

Our study had certain limitations. We detected correlations among the microflora, metabolites, and phenotypes in experimental mice under various feeding conditions; however, the causal relationships among these factors have not been clarified. In future studies, we plan to conduct population-based studies and evaluate the causal relationships.

Overall, NWLD combined the advantages of a variety of diets. It improved the abundance and composition of gut microbiota in obese mice, increased SCFA-producing bacteria, might increase SCFAs, and decreased the fecal metabolites that may have adverse effects on obesity. These changes in microflora and metabolites corrected obesity and metabolic disorders in mice. Novel weight loss diet shows potential as a novel dietary intervention program for obesity.

## Conclusions

This newly developed diet could effectively reduce body weight, optimize body composition, and reduce chronic inflammation and TG levels in a murine model of obesity. The phenotypic effects might be mediated by the NWLD-induced regulation of the intestinal microflora and metabolism. Novel weight loss diet attenuated disruptions in the intestinal microbiota. The bacterial communities with beneficial effects included *Desulphurvibrio, Ruminococcus, Bifidobacterium, Butyricicoccus, Gibbacter*, and *Gemmiger*. Metabolomics analyses illustrated that D-glutamine and D-glutamate, nitrogen, and histidine metabolic pathways, among which the key metabolites were L-aspartic acid and L-glutamic acid, were the key metabolic pathways altered by NWLD in HFD-fed mice. Although L-aspartic acid and L-glutamic acid may have adverse effects, our findings support the beneficial health effects of NWLD, providing a basis for further development of anti-obesity strategies aimed at optimizing a wide range of molecular and physiological factors.

## Patents

The customized dietary formula involved in this study has been patented (Application Number: 202111087743.3) and accepted by the China National Intellectual Property Administration (relevant information can be a query on http://cpquery.cnipa.gov.cn/), and intellectual property rights are protected.

## Data availability statement

The datasets presented in this study can be found in online repositories. The names of the repository/repositories and accession number(s) can be found below: Figshare, doi: 10.6084/m9.figshare.20462784.

## Ethics statement

The animal study was reviewed and approved by the Animal Care and Use Committee of the Scientific Research Ethics Committee, Beijing Shijitan Hospital, Capital Medical University (permission number: 2017-035).

## Author contributions

DY, XY, and YL: conceptualization. DY, XY, JL, and YL: methodology. XY and YC: software and visualization. XY, DY, YL, and HW: validation. XY: formal analysis and writing—original draft preparation. LB, YZ, and XY: investigation and data curation. DY: resources and funding acquisition. XY and DY: writing—review and editing and project administration. DY, JL, and XY: supervision. All authors have read and agreed to the published version of the manuscript.

## Funding

This research was funded by the Beijing Hospitals Authority' Ascent Plan, Code: DFL20190702.

## Conflict of interest

The authors declare that the research was conducted in the absence of any commercial or financial relationships that be construed as a potential conflict of interest.

## Publisher's note

All claims expressed in this article are solely those of the authors and do not necessarily represent those of their affiliated organizations, or those of the publisher, the editors and the reviewers. Any product that may be evaluated in this article, or claim that may be made by its manufacturer, is not guaranteed or endorsed by the publisher.

## References

[1] Seravalle G Grassi G. Obesity and hypertension. Pharmacol Res. (2017) 122:1–7. 10.1016/j.phrs.2017.05.013 28532816

[2] Iyengar NM Gucalp A Dannenberg AJ Hudis CA. Obesity and cancer mechanisms: tumor microenvironment and inflammation. J Clin Oncol. (2016) 34:4270–6. 10.1200/JCO.2016.67.4283 27903155 PMC5562428

[3] Dye L Boyle NB Champ C Lawton C. The relationship between obesity and cognitive health and decline. Proc Nutr Soc. (2017) 76:443–54. 10.1017/S0029665117002014 28889822

[4] Slavin JL. Dietary fiber and body weight. Nutrition. (2005) 21:411–8. 10.1016/j.nut.2004.08.018 15797686

[5] Sergeev IN Aljutaily T Walton G Huarte E. Effects of synbiotic supplement on human gut microbiota, body composition and weight loss in obesity. Nutrients. (2020) 12:222. 10.3390/nu12010222 31952249 PMC7019807

[6] Zhang J Wang H Wang Z Huang F Zhang X Du W. Trajectories of dietary patterns and their associations with overweight/obesity among Chinese adults: China Health and Nutrition Survey 1991–2018. Nutrients. (2021) 13:2835. 10.3390/nu13082835 34444995 PMC8401187

[7] Sainsbury E Kizirian NV Partridge SR Gill T Colagiuri S Gibson AA. Effect of dietary carbohydrate restriction on glycemic control in adults with diabetes: a systematic review and meta-analysis. Diabetes Res Clin Pract. (2018) 139:239–52. 10.1016/j.diabres.2018.02.026 29522789

[8] Gjuladin-Hellon T Davies IG Penson P Amiri Baghbadorani R. Effects of carbohydrate-restricted diets on low-density lipoprotein cholesterol levels in overweight and obese adults: a systematic review and meta-analysis. Nutr Rev. (2019) 77:161–80. 10.1093/nutrit/nuy049 30544168

[9] Meng Y Bai H Wang S Li Z Wang Q Chen L. Efficacy of low carbohydrate diet for type 2 diabetes mellitus management: a systematic review and meta-analysis of randomized controlled trials. Diabetes Res Clin Pract. (2017) 131:124–31. 10.1016/j.diabres.2017.07.006 28750216

[10] Bueno NB de Melo ISV de Oliveira SL da Rocha Ataide T. Very-low-carbohydrate ketogenic diet v. low-fat diet for long-term weight loss: a meta-analysis of randomised controlled trials. Br J Nutr. (2013) 110:1178–87. 10.1017/S0007114513000548 23651522

[11] Yu Z Nan F Wang LY Jiang H Chen W Jiang Y. Effects of high-protein diet on glycemic control, insulin resistance and blood pressure in type 2 diabetes: a systematic review and meta-analysis of randomized controlled trials. Clin Nutr. (2020) 39:1724–34. 10.1016/j.clnu.2019.08.008 31466731

[12] Schwingshackl L Hoffmann G. Long-term effects of low-fat diets either low or high in protein on cardiovascular and metabolic risk factors: a systematic review and meta-analysis. Nutr J. (2013) 12:48. 10.1186/1475-2891-12-48 23587198 PMC3636027

[13] Wycherley TP Moran LJ Clifton PM Noakes M Brinkworth GD. Effects of energy-restricted high-protein, low-fat compared with standard-protein, low-fat diets: a meta-analysis of randomized controlled trials. Am J Clin Nutr. (2012) 96:1281–98. 10.3945/ajcn.112.044321 23097268

[14] Johansson K Neovius M Hemmingsson E. Effects of anti-obesity drugs, diet, and exercise on weight-loss maintenance after a very-low-calorie diet or low-calorie diet: a systematic review and meta-analysis of randomized controlled trials. Am J Clin Nutr. (2014) 99:14–23. 10.3945/ajcn.113.070052 24172297 PMC3862452

[15] Barnard ND Levin SM Yokoyama Y. A systematic review and meta-analysis of changes in body weight in clinical trials of vegetarian diets. J Acad Nutr Diet. (2015) 115:954–69. 10.1016/j.jand.2014.11.016 25620754

[16] Yokoyama Y Levin SM Barnard ND. Association between plant-based diets and plasma lipids: a systematic review and meta-analysis. Nutr Rev. (2017) 75:683–98. 10.1093/nutrit/nux030 28938794 PMC5914369

[17] Huang RY Huang CC Hu FB Chavarro JE. Vegetarian diets and weight reduction: a meta-analysis of randomized controlled trials. J Gen Intern Med. (2016) 31:109–16. 10.1007/s11606-015-3390-7 26138004 PMC4699995

[18] Wang F Zheng J Yang B Jiang J Fu Y Li D. Effects of vegetarian diets on blood lipids: a systematic review and meta-analysis of randomized controlled trials. J Am Heart Assoc. (2015) 4:e002408. 10.1161/JAHA.115.002408 26508743 PMC4845138

[19] Davis C Bryan J Hodgson J Murphy K. Definition of the mediterranean diet; a literature review. Nutrients. (2015) 7:9139–53. 10.3390/nu7115459 26556369 PMC4663587

[20] Soenen S Bonomi AG Lemmens SG Scholte J Thijssen MA van Berkum F. Relatively high-protein or ‘low-carb' energy-restricted diets for body weight loss and body weight maintenance? Physiol Behav. (2012) 107:374–80. 10.1016/j.physbeh.2012.08.004 22935440

[21] Bilsborough SA Crowe TC. Low-carbohydrate diets: what are the potential short- and long-term health implications? Asia Pac J Clin Nutr. (2003) 12:396–404. 14672862

[22] Tovar-Palacio C Tovar AR Torres N Cruz C Hernández-Pando R Salas-Garrido G. Proinflammatory gene expression and renal lipogenesis are modulated by dietary protein content in obese Zucker^fa/fa^ rats. Am J Physiol Renal Physiol. (2011) 300:F263–71. 10.1152/ajprenal.00171.2010 20962115

[23] Neufingerl N Eilander A. Nutrient intake and status in adults consuming plant-based diets compared to meat-eaters: a systematic review. Nutrients. (2021) 14:29. 10.3390/nu14010029 35010904 PMC8746448

[24] Kiely ME. Risks and benefits of vegan and vegetarian diets in children. Proc Nutr Soc. (2021) 80:159–64. 10.1017/S002966512100001X 33504371

[25] Estruch R Ros E. The role of the Mediterranean diet on weight loss and obesity-related diseases. Rev Endocr Metab Disord. (2020) 21:315–27. 10.1007/s11154-020-09579-0 32829455

[26] Wan Y Wang F Yuan J Li J Jiang D Zhang J. Effects of macronutrient distribution on weight and related cardiometabolic profile in healthy non-obese Chinese: a 6-month, randomized controlled-feeding trial. EBioMedicine. (2017) 22:200–7. 10.1016/j.ebiom.2017.06.017 28655596 PMC5672080

[27] Chinese Residents' Nutrition Chronic Disease. (2020). Available online at: http://www.gov.cn/xinwen/2020-12/24/content_5572983.htm

[28] David LA Maurice CF Carmody RN Gootenberg DB Button JE Wolfe BE. Diet rapidly and reproducibly alters the human gut microbiome. Nature. (2014) 505:559–63. 10.1038/nature12820 24336217 PMC3957428

[29] Maier L Pruteanu M Kuhn M Zeller G Telzerow A Anderson EE. Extensive impact of non-antibiotic drugs on human gut bacteria. Nature. (2018) 555:623–8. 10.1038/nature25979 29555994 PMC6108420

[30] Asnicar F Berry SE Valdes AM Nguyen LH Piccinno G Drew DA. Microbiome connections with host metabolism and habitual diet from 1,098 deeply phenotyped individuals. Nat Med. (2021) 27:321–32. 10.1038/s41591-020-01183-8 33432175 PMC8353542

[31] Jian C Silvestre MP Middleton D Korpela K Jalo E Broderick D. Gut microbiota predicts body fat change following a low-energy diet: a preview intervention study. Genome Med. (2022) 14:54. 10.1186/s13073-022-01053-7 35599315 PMC9125896

[32] Dong TS Luu K Lagishetty V Sedighian F Woo SL Dreskin BW. A high protein calorie restriction diet alters the gut microbiome in obesity. Nutrients. (2020) 12:3221. 10.3390/nu12103221 33096810 PMC7590138

[33] Rinott E Meir AY Tsaban G Zelicha H Kaplan A Knights D. The effects of the Green-Mediterranean diet on cardiometabolic health are linked to gut microbiome modifications: a randomized controlled trial. Genome Med. (2022) 14:29. 10.1186/s13073-022-01015-z 35264213 PMC8908597

[34] Benítez-Páez A Hess AL Krautbauer S Liebisch G Christensen L Hjorth MF. Sex, food, and the gut microbiota: disparate response to caloric restriction diet with fiber supplementation in women and men. Mol Nutr Food Res. (2021) 65:e2000996. 10.1002/mnfr.202000996 33629506

[35] Wan Y Wang F Yuan J Li J Jiang D Zhang J. Effects of dietary fat on gut microbiota and faecal metabolites, and their relationship with cardiometabolic risk factors: a 6-month randomised controlled-feeding trial. Gut. (2019) 68:1417–29. 10.1136/gutjnl-2018-317609 30782617

[36] Zhao S Kang R Deng T Luo L Wang J Li E. Comparison of two cannulation methods for assessment of intracavernosal pressure in a rat model. PLoS ONE. (2018) 13:e0193543. 10.1371/journal.pone.0193543 29486011 PMC5828359

[37] Callahan BJ McMurdie PJ Rosen MJ Han AW Johnson AJ Holmes SP. DADA2: high-resolution sample inference from Illumina amplicon data. Nat Methods. (2016) 13:581–3. 10.1038/nmeth.3869 27214047 PMC4927377

[38] Bokulich NA Kaehler BD Rideout JR Dillon M Bolyen E Knight R. Optimizing taxonomic classification of marker-gene amplicon sequences with QIIME 2's q2-feature-classifier plugin. Microbiome. (2018) 6:90. 10.1186/s40168-018-0470-z 29773078 PMC5956843

[39] Love MI Huber W Anders S. Moderated estimation of fold change and dispersion for RNA-seq data with DESeq2. Genome Biol. (2014) 15:550. 10.1186/s13059-014-0550-8 25516281 PMC4302049

[40] Segata N Izard J Waldron L Gevers D Miropolsky L Garrett WS. Metagenomic biomarker discovery and explanation. Genome Biol. (2011) 12:R60. 10.1186/gb-2011-12-6-r60 21702898 PMC3218848

[41] Mandal S Van Treuren W White RA Eggesbø M Knight R Peddada SD. Analysis of composition of microbiomes: a novel method for studying microbial composition. Microb Ecol Health Dis. (2015) 26:27663. 10.3402/mehd.v26.27663 26028277 PMC4450248

[42] Vázquez-Baeza Y Pirrung M Gonzalez A Knight R. EMPeror: a tool for visualizing high-throughput microbial community data. Gigascience. (2013) 2:16. 10.1186/2047-217X-2-16 24280061 PMC4076506

[43] Langille MG Zaneveld J Caporaso JG McDonald D Knights D Reyes JA. Predictive functional profiling of microbial communities using 16S rRNA marker gene sequences. Nat Biotechnol. (2013) 31:814–21. 10.1038/nbt.2676 23975157 PMC3819121

[44] Chong J Xia J. MetaboAnalystR: an R package for flexible and reproducible analysis of metabolomics data. Bioinformatics. (2018) 34:4313–4. 10.1093/bioinformatics/bty528 29955821 PMC6289126

[45] Cândido FG Valente FX Grześkowiak ŁM Moreira APB Rocha D Alfenas RCG. Impact of dietary fat on gut microbiota and low-grade systemic inflammation: mechanisms and clinical implications on obesity. Int J Food Sci Nutr. (2018) 69:125–43. 10.1080/09637486.2017.1343286 28675945

[46] Dongowski G Lorenz A Anger H. Degradation of pectins with different degrees of esterification by *Bacteroides thetaiotaomicron* isolated from human gut flora. Appl Environ Microbiol. (2000) 66:1321–7. 10.1128/AEM.66.4.1321-1327.2000 10742206 PMC91987

[47] Jensen NS Canale-Parola E. *Bacteroides pectinophilus* sp. nov. and *Bacteroides galacturonicus* sp. nov.: two pectinolytic bacteria from the human intestinal tract. Appl Environ Microbiol. (1986) 52:880–7. 10.1128/aem.52.4.880-887.1986 3777933 PMC239131

[48] Koropatkin NM Cameron EA Martens EC. How glycan metabolism shapes the human gut microbiota. Nat Rev Microbiol. (2012) 10:323–35. 10.1038/nrmicro2746 22491358 PMC4005082

[49] Flint A Raben A Rehfeld JF Holst JJ Astrup A. The effect of glucagon-like peptide-1 on energy expenditure and substrate metabolism in humans. Int J Obes Relat Metab Disord. (2000) 24:288–98. 10.1038/sj.ijo.0801126 10757621

[50] Batterham RL Cowley MA Small CJ Herzog H Cohen MA Dakin CL. Gut hormone PYY(3-36) physiologically inhibits food intake. Nature. (2002) 418:650–4. 10.1038/nature00887 12167864

[51] Holz GG. Habener JF. Pancreatic beta-cells are rendered glucose-competent by the insulinotropic hormone glucagon-like peptide-1(7-37). Nature. (1993) 361:362–5. 10.1038/361362a0 8381211 PMC2916679

[52] Corrales P Vidal-Puig A Medina-Gómez G. PPARs and metabolic disorders associated with challenged adipose tissue plasticity. Int J Mol Sci. (2018) 19:2124. 10.3390/ijms19072124 30037087 PMC6073677

[53] Windmueller HG Spaeth AE. Metabolism of absorbed aspartate, asparagine, and arginine by rat small intestine *in vivo*. Arch Biochem Biophys. (1976) 175:670–6. 10.1016/0003-9861(76)90558-0 958325

[54] Moszak M Klupczyńska A Kanikowska A Kokot Z Zawada A Grzymisławska M. The influence of a 3-week body mass reduction program on the metabolic parameters and free amino acid profiles in adult Polish people with obesity. Adv Clin Exp Med. (2018) 27:749–57. 10.17219/acem/70796 29790679

[55] Takashina C Tsujino I Watanabe T Sakaue S Ikeda D Yamada A. Associations among the plasma amino acid profile, obesity, and glucose metabolism in Japanese adults with normal glucose tolerance. Nutr Metab (Lond). (2016) 13:5. 10.1186/s12986-015-0059-5 26788116 PMC4717594

[56] Maltais-Payette I Allam-Ndoul B Pérusse L Vohl MC Tchernof A. Circulating glutamate level as a potential biomarker for abdominal obesity and metabolic risk. Nutr Metab Cardiovasc Dis. (2019) 29:1353–60. 10.1016/j.numecd.2019.08.015 31668457

[57] Matyšková R Maletínská L Maixnerová J Pirník Z Kiss A Železná B. Comparison of the obesity phenotypes related to monosodium glutamate effect on arcuate nucleus and/or the high fat diet feeding in C57BL/6 and NMRI mice. Physiol Res. (2008) 57:727–34. 10.33549/physiolres.931274 17949248

[58] Lee WJ Hawkins RA Viña JR Peterson DR. Glutamine transport by the blood-brain barrier: a possible mechanism for nitrogen removal. Am J Physiol. (1998) 274:C1101–7. 10.1152/ajpcell.1998.274.4.C1101 9580550

[59] Zapata RC Singh A Pezeshki A Chelikani PK. Tryptophan restriction partially recapitulates the age-dependent effects of total amino acid restriction on energy balance in diet-induced obese rats. J Nutr Biochem. (2019) 65:115–27. 10.1016/j.jnutbio.2018.12.006 30685580

[60] Kimura K Nakamura Y Inaba Y Matsumoto M Kido Y Asahara S. Histidine augments the suppression of hepatic glucose production by central insulin action. Diabetes. (2013) 62:2266–77. 10.2337/db12-1701 23474485 PMC3712067

[61] Bellissimo MP Cai Q Ziegler TR Liu KH Tran PH Vos MB. Plasma high-resolution metabolomics differentiates adults with normal weight obesity from lean individuals. Obesity (Silver Spring). (2019) 27:1729–37. 10.1002/oby.22654 31689010 PMC6839782

[62] Li R Huang X Liang X Su M Lai KP Chen J. Integrated omics analysis reveals the alteration of gut microbe-metabolites in obese adults. Brief Bioinform. (2021) 22:bbaa165. 10.1093/bib/bbaa165 32770198

