# Supplementary material for: Novel weight loss diet attenuates dietary-induced obesity in mice and might correlate with altered gut microbiota and metabolite profiles
Source: Front Nutr. 2022 Nov 11;9:987955. doi: 10.3389/fnut.2022.987955 (PMC9692001; doi:10.3389/fnut.2022.987955)
Supplement: Supplementary file 1 [file Data_Sheet_1.PDF]

Supplementary Material

1 Supplementary Method

16S rRNA Gene Sequencing Analysis

The PCR reactions were conducted using the following program: 3 min of denaturation at 95 °C, 27 cycles of 30 s at 95 °C, 30s for annealing at 55 °C, and 45s for elongation at 72 °C, and a final extension at 72 °C for 10 min. PCR reactions were performed in triplicate 20 μL mixture containing 4 μL of 5 × FastPfu Buffer, 2 μL of 2.5 mM dNTPs, 0.8 μL of each primer (5 μM), 0.4 μL of FastPfu Polymerase and 10 ng of template DNA. The resulted PCR products were extracted from a 2% agarose gel and further purified using the AxyPrep DNA Gel Extraction Kit (Axygen Biosciences, Union City, CA, USA) and quantified using QuantiFluor™-ST (Promega, USA) according to the manufacturer's protocol. Illumina NovaSeq sequencing Purified amplicons were pooled in equimolar and paired-end sequenced (2 × 250) on an Illumina NovaSeq platform (Illumina, San Diego, USA) according to the standard protocols.

Fecal Metabolic Analysis

Quality control sample   As a part of the system conditioning and quality control process, a pooled quality control sample (QC) was prepared by mixing equal volumes of all samples. The QC samples were disposed and tested in the same manner as the analytic samples. It helped to represent the whole sample set, which would be injected at regular intervals (every 3 samples) in order to monitor the stability of the analysis.

Ultra-Performance liquid chromatography/tandem mass spectrometry (UPLC-MS/MS) analysis Metabolites were profiled using a UPLC-Triple-TOF-MS-based platform.

Chromatographic separation of the metabolites was performed on a ExionLCTMAD system (AB Sciex, USA) equipped with an ACQUITY UPLC BEH C18 column (100 mm × 2.1 mm i.d., 1.7 μm; Waters, Milford, USA). The mobile phases consisted of 0.1% formic acid in water with formic acid (0.1%) (solvent A) and 0.1% formic acid in acetonitrile: isopropanol (1:1, v/v) (solvent B). The solvent gradient changed according to the following conditions: from 0 to 3 min, 95% (A): 5% (B) to 80% (A): 20% (B); from 3 to 9 min, 80% (A): 20% (B) to 5% (A): 95% (B); from 9 to 13 min, 5% (A): 95% (B) to 5% (A): 95% (B); from 13 to 13.1 min, 5% (A): 95% (B) to 95% (A): 5% (B), from 13.1 to 16 min, 95% (A): 5% (B) to 95% (A): 5% (B) for equilibrating the systems. The sample injection volume was 20μL and the flow rate was set to 0.4 mL/min. The column temperature was maintained at 40 °C. During the period of analysis, all these samples were stored at 4 °C.

The UPLC system was coupled to a quadrupole-time-of-flight mass spectrometer (Triple TOFTM5600+, AB Sciex, USA) equipped with an electrospray ionization (ESI) source operating in positive mode and negative mode. The optimal conditions were set as followed: source temperature, 500 °C; curtain gas (CUR), 30 psi; both Ion Source GS1 and GS2, 50 psi; ion-spray voltage floating (ISVF), -4000V in negative mode and 5000V in positive mode, respectively; declustering potential, 80V; a collision energy (CE), 20-60V rolling for MS/MS. Data acquisition was performed with the Data Dependent Acquisition (DDA) mode. The detection was carried out over a mass range of 50-1000 m/z.

After UPLC-TOF/MS analyses, the raw data were imported into the Progenesis QI 2.3 (Nonlinear Dynamics, Waters, USA) for peak detection and alignment. The preprocessing results generated a data matrix that consisted of the retention time (RT), mass-to-charge ratio (m/z) values, and peak intensity. After filtering, minimum metabolite values were imputed for specific samples in which the metabolite levels fell below the lower limit of quantitation and each Metabolic features were normalized by sum. The internal standard was used for data QC (reproducibility), Metabolic features which the relative standard deviation (RSD) of QC >30% were discarded. In order to identify significant differences in metabolite levels between comparable groups, statistical analyzed according to normalization procedures and normalization to determine significant differences in metabolite levels between comparable groups. Mass spectra of these metabolic features were identified by using the accurate mass, MS/MS fragments spectra and isotope ratio difference with searching in reliable biochemical databases as Human metabolome database (HMDB) (http://www.hmdb.ca/) and Metlin database (https://metlin.scripps.edu/). Concretely, the mass tolerance between the measured m/z values and the exact mass of the components of interest was ± 5ppm. For metabolites having MS/MS confirmation, only the ones with MS/MS fragments score above 30 were considered as confidently identified. Otherwise, metabolites had only tentative assignments.

2 Supplementary Figures

Figure 1

Figure 1. Comparison of phenotypic factors among groups. Bar charts of (**A**) serum triglycerides (TG), (**B**) hs-CRP, (**C**) IL-1β, (**D**) TNF-α, **(E)** total fat, (**F**) subcutaneous fat percentages, (**G**) visceral fat percentages, (**H**) brown fat percentages, and (**I**) white fat (the highlighted areas in the abdomen and subcutaneous tissue) in each group (coronal plane). Images were taken from NC4 section 7, HFD3 section 9, and NWLD3 section 6. (**J**) Brown fat of mice in different groups (butterfly areas are indicated by arrows). Images were taken from NC2 section 8, 5 section 29, and NWLD4 section 29. * $p < 0.05$; ** $p < 0.01$; *** $p < 0.001$; **** $p < 0.0001$. Bar charts of (K) serum creatinine, (L) serum urea, (M) renal index.

Figure 2

Figure 2. Characteristics of the gut microbiota in different groups. (**A**) histograms of the relative distribution of groups at the phylum level (top 20 species in relative abundance); (**B**) histograms of the relative distribution of groups at the genus level (top 20 species in

relative abundance); (**C**) alpha diversity. Box plots of observed OTUs, histogram of Chao1, Shannon, and Simpson indices. ANOVA, * $p < 0.05$; ns, no significance. (**D**) Beta diversity; (**E**) LEfSe analysis LDA histograms (LDA score >2.0) and cladogram of characteristic microorganisms (group NC compared to group HFD). Kruskal–Wallis test, * $p < 0.05$. LDA, each transverse column represents a species; the length of the column represents the LDA score, where a higher LDA score indicates a greater difference. The color of the bar indicates the species group. Layers from the inside to outside of the cladogram correspond to different classification levels, i.e., kingdom, phylum, class, order, family, and genus, and the lines between levels represent relationships. Each circular node represents a species. Yellow nodes indicate no significant difference between groups; non-yellow nodes indicate that the species is a characteristic microorganism of the corresponding group (with significantly higher abundance in this group). The shaded fans mark the subclassification interval of the characteristic microorganism. (**F**) LEfSe analysis LDA histograms (LDA score >2.0) and cladogram of characteristic microorganisms (group NWLD compared to group HFD). Kruskal–Wallis test, * $p < 0.05$.

Figure 3

Figure 3. Fecal untargeted metabolomics results. (**A**) principal component analysis (PCA) score plots for discriminating the fecal metabolome (positive and negative charge metabolites) from the NC, HFD, and NWLD groups; (**B**) SVM ROC curves. The abscissa indicates accuracy, and the ordinate indicates sensitivity. AUC values closer to 1 indicate a better predictive accuracy and a greater difference in metabolites between groups; (**C**) ORA and topology analyses of metabolic pathways with significant differences between groups NC and HFD. The horizontal coordinate represents the ORA p-value, and the blue area is significant ($p < 0.05$). The vertical axis shows the impact value in the topology analysis. Maps of the main metabolic pathways were shown, including the alanine, aspartate, and glutamate metabolism pathways and steroid hormone biosynthesis; (**D**) ORA and topological analyses of the metabolic pathways with significant differences between groups NWLD and HFD and maps of the main metabolic pathways, including D-glutamine, D-glutamate, nitrogen, and histidine metabolism pathways;

Attached figures

Interaction network of phenotypic factors, gut microbes at the genus level, and fecal metabolites (Spearman correlation analysis). Circles represent a species, the size of each circle represents the relative abundance, and different colors represent different phyla. The lines between the circles represent significant correlations between the relative frequencies of the two species ($p < 0.05$). The red and blue lines represent positive and negative correlations, respectively.

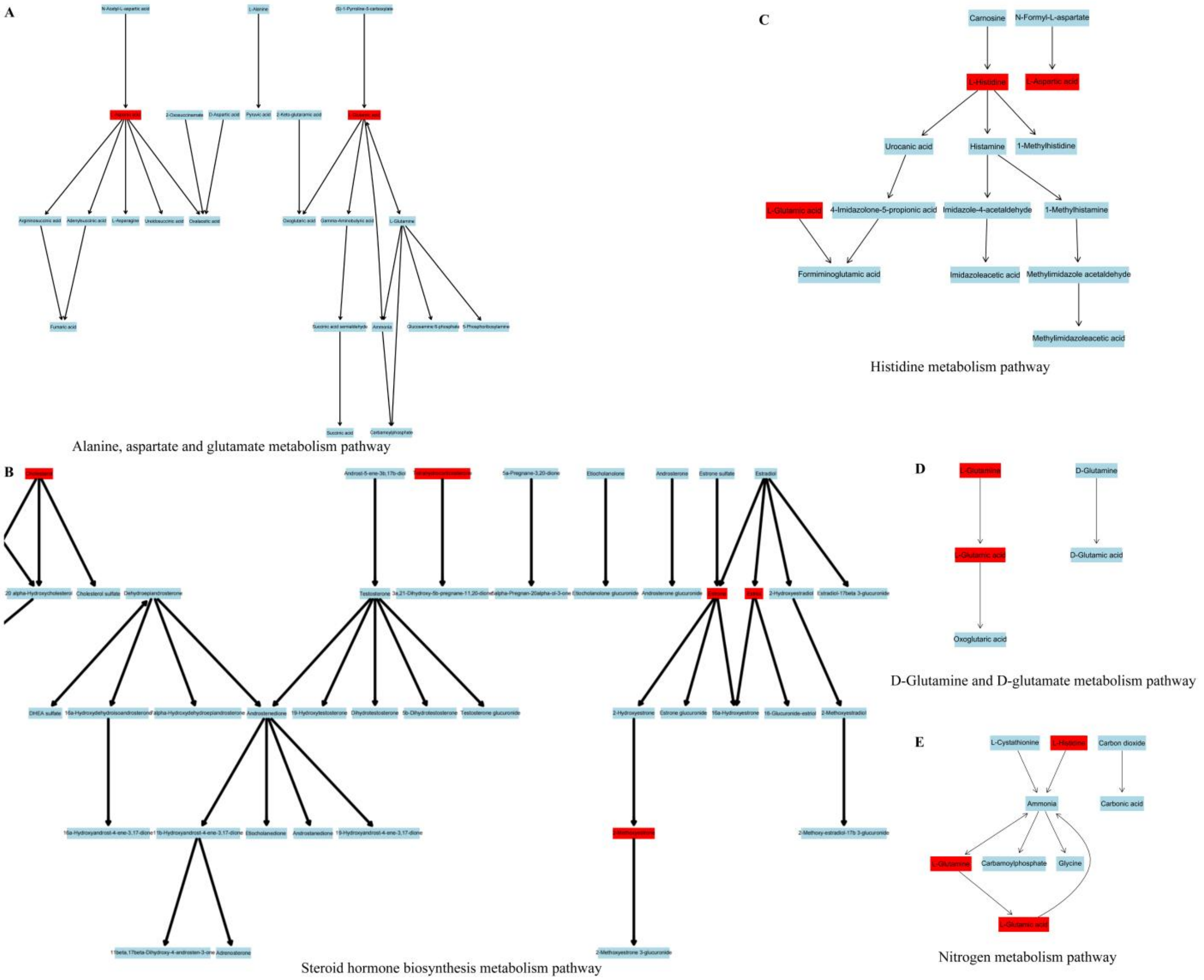

Attached figure 1. Metabolite differences were concentrated in the comparisons between groups NC and HFD: (**A**) Alanine,aspartate and glutamate metabolism pathway; (**B**) Steroid hormone biosynthesis; Metabolite differences were concentrated in the comparisons between groups NWLD and HFD: (**C**) Histidine metabolism pathway; (**D**) D-Glutamine and D-glutamate metabolism pathway; **(E**) Nitrogen metabolism pathway.

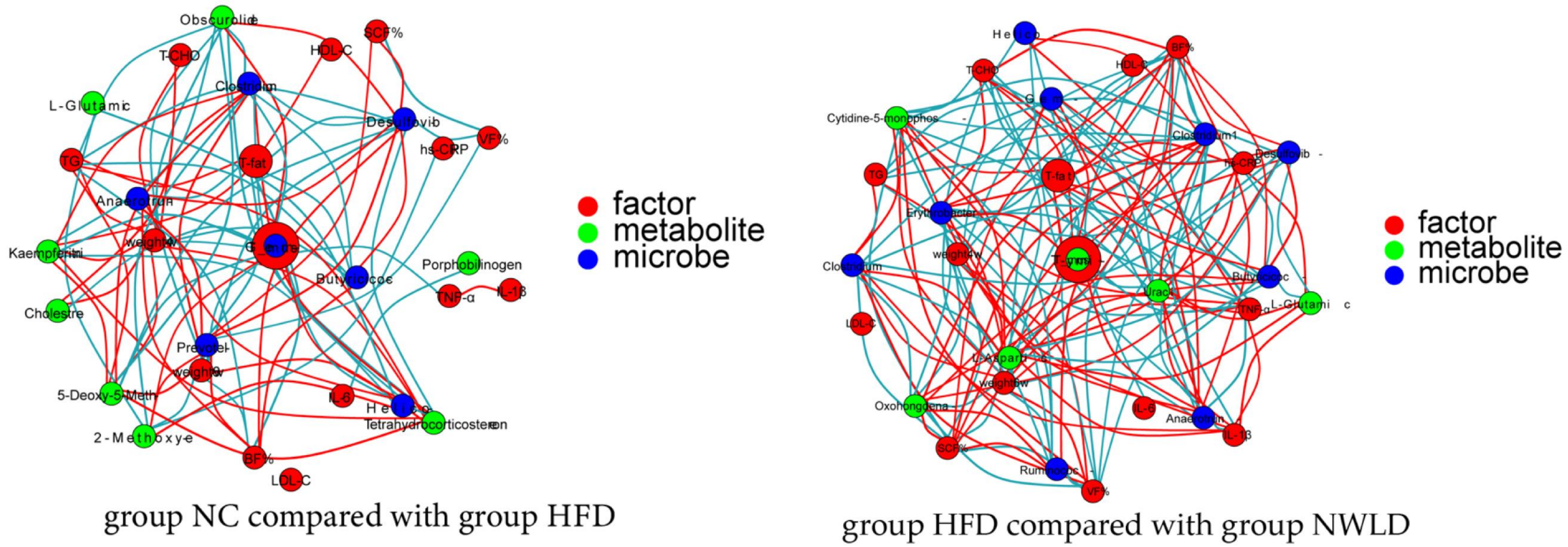

T-fat, total fat; TG, total cholesterol; hs-CRP, high-sensitivity C-reactive protein; TNF-α, tumor necrosis factor-α; IL-1β, interleukin-1β; VF%, visceral fat percentage; BF%, brown fat percentage; SCF%, subcutaneous fat percentage ; T-CHO .T-muscle, total muscle; T-fat,total fat.

Attached figure 2. Interaction network of phenotypic factors, gut microbes at the genus level, and fecal metabolites (Spearman correlation analysis). Circles represent a species, the size of each circle represents the relative abundance, and different colors represent different phyla. The lines between the circles represent significant correlations between the relative frequencies of the two species ($p < 0.05$). The red and blue lines represent positive and negative correlations, respectively.
